# Supplementary material for: Comparative multiomics analysis of cell physiological state after culture in a basket bioreactor
Source: Sci Rep. 2022 Nov 23;12:20161. doi: 10.1038/s41598-022-24687-4 (PMC9686226; doi:10.1038/s41598-022-24687-4)
Supplement: Supplementary file 1 — Supplementary Information 1. [file 41598_2022_24687_MOESM1_ESM.zip › raw data/Transcriptomic raw data/6.Enrichment/2.KEGG/TE1vsTC1/GSEA/BIOSYNTHESIS_OF_AMINO_ACIDS(CSAB01230).html]

Details for gene set BIOSYNTHESIS\_OF\_AMINO\_ACIDS(CSAB01230)[GSEA]

|  || Dataset | readcount.sample |
| Phenotype | sample.cls |
| Upregulated in class | TE1 |
| GeneSet | BIOSYNTHESIS\_OF\_AMINO\_ACIDS(CSAB01230) |
| Enrichment Score (ES) | 0.93588066 |
| Normalized Enrichment Score (NES) | 1.3017544 |
| Nominal p-value | 0.0 |
| FDR q-value | 0.49185848 |
| FWER p-Value | 0.49 |
Table: GSEA Results Summary

  

Fig 1: Enrichment plot: BIOSYNTHESIS\_OF\_AMINO\_ACIDS(CSAB01230)      
 Profile of the Running ES Score & Positions of GeneSet Members on the Rank Ordered List

  

| PROBE | DESCRIPTION (from dataset) | GENE SYMBOL | GENE\_TITLE | RANK IN GENE LIST | RANK METRIC SCORE | RUNNING ES | CORE ENRICHMENT || 1 | novel.572 | - |  |  | 628 | 10.870 | 0.9359 | Yes |
| 2 | novel.266 | - |  |  | 18854 | 0.407 | 0.1584 | No |
Table: GSEA details [plain text format]

  

Fig 2: BIOSYNTHESIS\_OF\_AMINO\_ACIDS(CSAB01230)      
 Blue-Pink O' Gram in the Space of the Analyzed GeneSet

  

Fig 3: BIOSYNTHESIS\_OF\_AMINO\_ACIDS(CSAB01230): Random ES distribution      
 Gene set null distribution of ES for **BIOSYNTHESIS\_OF\_AMINO\_ACIDS(CSAB01230)**
